# Economic Evaluation of Internet-Based Interventions for Harmful Alcohol Use Alongside a Pragmatic Randomized Controlled Trial

**DOI:** 10.2196/jmir.2052

**Published:** 2012-10-29

**Authors:** Matthijs Blankers, Udo Nabitz, Filip Smit, Maarten WJ Koeter, Gerard M Schippers

**Affiliations:** ^1^Department JellinekArkin Mental Health CareAmsterdamNetherlands; ^2^Amsterdam Institute for Addiction ResearchDepartment of PsychiatryAcademic Medical Centre, University of AmsterdamAmsterdamNetherlands; ^3^Trimbos InstituteNetherlands Institute of Mental Health and AddictionUtrechtNetherlands; ^4^EMGO InstituteDepartment of Epidemiology and BiostatisticsVU University Medical CentreAmsterdamNetherlands

**Keywords:** Cost-benefit analysis, randomized controlled trial, alcohol-induced disorders, self-help, computer-assisted therapy

## Abstract

**Background:**

Internet interventions with and without therapist support have been found to be effective treatment options for harmful alcohol users. Internet-based therapy (IT) leads to larger and longer-lasting positive effects than Internet-based self-help (IS), but it is also more costly to provide.

**Objective:**

To evaluate the cost effectiveness and cost utility of Internet-based interventions for harmful use of alcohol through the assessment of the incremental cost effectiveness of IT compared with IS.

**Methods:**

This study was performed in a substance abuse treatment center in Amsterdam, the Netherlands. We collected data over the years 2008–2009. A total of 136 participants were included, 70 (51%) were female, and mean age was 41.5 (SD 9.83) years. Reported alcohol consumption and Alcohol Use Disorders Identification Test (AUDIT) scores indicated harmful drinking behavior at baseline. We collected self-reported outcome data prospectively at baseline and 6 months after randomization. Cost data were extracted from the treatment center’s cost records, and sex- and age-specific mean productivity cost data for the Netherlands.

**Results:**

The median incremental cost-effectiveness ratio was estimated at €3683 per additional treatment responder and €14,710 per quality-adjusted life-year (QALY) gained. At a willingness to pay €20,000 for 1 additional QALY, IT had a 60% likelihood of being more cost effective than IS. Sensitivity analyses attested to the robustness of the findings.

**Conclusions:**

IT offers better value for money than IS and might therefore be considered as a treatment option, either as first-line treatment in a matched-care approach or as a second-line treatment in the context of a stepped-care approach.

**Trial Registration:**

Netherlands Trial Register NTR-TC1155; http://www.trialregister.nl/trialreg/admin/rctview.asp?TC=1155 (Archived by WebCite at http://www.webcitation.org/6AqnV4eTU)

## Introduction

Harmful alcohol use is the number-3 leading contributor to global burden of disease [1] and causes 3.8% of global mortality [2], as well as losses in gross domestic product [3]. The majority of people with alcohol use disorders are not receiving any form of treatment, leading to a treatment gap [4]. Among the possible means of bridging this treatment gap is the use of accessible and efficient treatment, delivered over the Internet. Internet interventions with and without therapist support [5-8] have been found to be effective treatment options for harmful alcohol users and could perhaps be used sequentially in a stepped-care format. Internet-based therapy (IT) leads to larger and longer-lasting positive effects than Internet-based self-help (IS) in the treatment of depression [9,10], anxiety [10,11], and problem drinking [8]. However, IT is more costly to provide and more demanding for both participants and therapists. Thus, the research question is, “Does the additional positive result of therapist support outweigh its additional cost?” We present an economic evaluation assessing the cost effectiveness and cost utility of IT compared with IS for harmful alcohol use. Recently, studies have been published on the cost effectiveness of Internet-based (self-help) interventions for depression [12], weight management [13], and harmful alcohol use [14]. The cost effectiveness of therapist support in Internet-based alcohol interventions has not yet been supported, however.

## Methods

### Study Design and Participants

We collected data for the cost effectiveness analysis alongside a pragmatic randomized controlled trial on the effectiveness of IT relative to IS and a waiting list, conducted in the Netherlands in 2008–2009. Because in economic evaluation the preferred comparison is between the intervention of interest (IT) and its best alternative, in this case IS, we do not present waiting list data in this paper.

**Figure 1:**
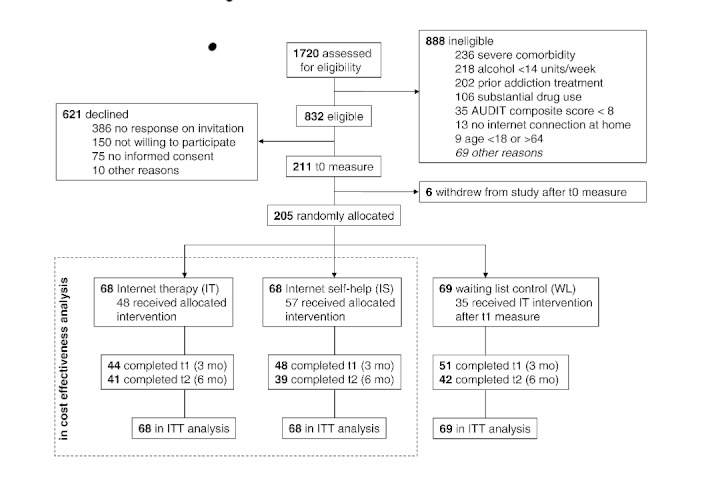
CONSORT trial flow diagram for the randomized controlled trial. AUDIT = Alcohol Use Disorders Identification Test, ITT = intention-to-treat analysis, IS = Internet-based self-help, IT = Internet-based therapy.

We recruited applicants through jellinek.nl, a substance abuse treatment center website with 650,000 visitors annually [8]. For inclusion, applicants had to (1) be between 18 and 65 years old, (2) live in the Netherlands with health care insurance coverage, (3) have Internet access at home, (4) score above 8 on the Alcohol Use Disorders Identification Test (AUDIT) [15], (5) report a weekly consumption of more than 14 standard (10 g ethanol) drinking units, and (6) provide informed consent. Exclusion criteria were (1) prior substance abuse treatment, (2) a history of alcohol delirium or drug overdose, (3) a history of severe cardiovascular or gastrointestinal diseases, (4) a history of schizophrenia, epilepsy, or suicidal tendencies, (5) extensive substance use in the last month, and (6) unavailability of more than 2 weeks during the study. Of the 1720 who were assessed, 832 applicants were eligible for inclusion; 205 participants were included. Compared with all 832 eligible applicants, the 205 included participants reported somewhat higher baseline AUDIT scores, but this difference was not significant (mean 18.9, SD 4.98 vs mean 19.5, SD 5.13, *t*_204 _= 1.617, *P *= .11). In the IT group, 48 received the allocated intervention, that is, they participated in treatment exercises and chat therapy. In IS, 57 received the intervention, which consisted of exercises only. Outcome data were collected at baseline, 3 months, and 6 months after randomization (Figure 1). The study design [16] and outcomes of the randomized controlled trial [8] were published elsewhere.

### Interventions

Both IT and IS were based on a cognitive behavioral therapy and motivational interviewing treatment protocol [17]. In IS, participants were introduced to various treatment exercises. Without a therapist’s support, participants acquired skills and knowledge about coping with craving, drinking lapses, and peer pressure. IT was driven by 7 synchronous text-based chat-therapy sessions with a personal (Internet) cognitive behavioral therapy-trained therapist, lasting 40 minutes each, and accompanied by homework assignments. Each of the chat-therapy sessions had its own theme: monitoring and goal setting, self-control, and relapse prevention, for example.

### Cost Measures

In this economic evaluation, we used the societal perspective. All costs related to IT and IS interventions, health care uptake, opportunity costs of the participant’s time, and productivity losses were included. All costs (Table 1) are expressed in euros and were indexed to the reference year 2010 using an inflation correction based on the Harmonized Index of Consumer Prices (HICP) [18].

**Table 1:** Unit costs and average quantities per participant for Internet-based therapy and Internet-based self-help.

Cost type		Unit	Internet therapy		Internet self-help	
			No. of units	€/unit	No. of units	€/unit	
**Intervention costs**							
	Therapist therapy	Hour	2.49	79.20	NA^a^	NA
	Therapist administration	Hour	0.55	79.20	NA	NA
	Software development	Participant	1	23.25	1	4.87
	ICT^b ^service	Participant	1	14.92	1	2.49
	Software overhead	Participant	1	4.27	1	4.27
	Total intervention costs	Participant	1	283.21^c^	1	11.63^c^
Participant’s leisure time		Hour	10.33	9.18	2.43	9.18
Work absenteeism^d^		Hour	32.12	22.21–52.91^e^	18.35	22.21–52.91^e^
Work presenteeism^f^		Hour	8.15	22.21–52.91^e^	12.15	22.21–52.91^e^

^a ^Not applicable.

^b ^Information and computer technology.

^c ^Average intervention cost per participant. Individual costs varied and depended on the amount of intervention uptake.

^d ^Average number of work hours lost in the 6 months preceding measurement due to participants not going to work (eg, sick leave).

^e ^Range of unit cost. The unit value was dependent on sex and age of the participant and based on 2010 Harmonized Index of Consumer Prices inflation-corrected average hourly wages [19].

^f ^Average number of work hours lost in the 6 months preceding measurement due to participants not functioning well professionally while at work.

IT and IS intervention costs consisted of software development costs, information and computer technology service costs, overhead costs (based on the treatment center’s cost records), and—for IT only—therapist-related costs. We collected the cost data over the years 2004-2009. Information and computer technology service costs were based on averaged annual costs and included server rental costs, software security costs, and a monthly information and computer technology support fee. Overhead costs were based on actual time investment estimations. Time invested was multiplied by labor costs based on collective labor agreement wages, with 50% additional employer costs for overhead and insurance. Development, information and computer technology service, and overhead costs were divided by the monthly recorded number of participants (IT: 25; IS: 50). Therapist costs were based on the actual chat-contact time, with an added 10 minutes per chat session for supervision and administrative work. Therapist work time was valued based on average sex-, age-, and profession-specific labor costs in the Netherlands [19], which resulted in €1.32 per minute in 2010. It is worth noting that the cost price for labor was in line with the costs (€80) for a single contact session with a primary care psychologist in the Netherlands in 2009 [20].

We restricted participant costs to a valuation of their time investment, valued as leisure time at €9.18 per hour [19], assuming that the therapy was not received during their office hours. Time investment for participants per treatment session, including homework, was 20 minutes (based on user inquiry) plus the therapy duration in the case of IT chat therapy. We collected data on productivity losses stemming from absenteeism and presenteeism using the Short Form-Health and Labor Questionnaire (SF-HLQ), a subscale of the Trimbos/iMTA questionnaire for Costs associated with Psychiatric illness [21]. Productivity costs were collected over a 2-week period before data collection, in correspondence with the SF-HLQ manual [21]. The reported costs over this 2-week period were then extrapolated. This method was found to be valid in patients with cluster B personality disorders [22], but we did not validate it in the current population of harmful alcohol users. To value inefficient job performance, these data were combined with sex- and age-specific mean productivity cost data for the Netherlands [20]. We used an elasticity estimate of 0.8, as suggested by the Netherlands Economic Institute [23]; we assumed that in case of absence, 20% of the production had not been lost but was compensated for by a firm’s internal labor reserves. Considering the limited time horizon of collected cost data in this study, duration of absenteeism was valued according to the human capital approach. We therefore regarded cost as accrued for the full period of absenteeism and not limited to a friction period [24].

Additional societal costs were calculated using a macroscopic approach based on global burden of disease and injury data [2]. For high-income countries, productivity losses are the primary contributor to total alcohol-attributable costs: productivity loss accounts for 72.1% of the overall societal costs [2]. Additional health care resource costs (12.8%) and law-enforcement costs (3.5%) were estimated based on productivity cost data. Costs due to property damage, administration, or social work services were not taken into account, as these costs are excluded in most economic evaluations. The timeframe for this study and all time-variant costs was 6 months.

### Effect Measures

The central clinical outcome for the cost effectiveness analysis was treatment response, based on alcohol consumption during the last 7 days. In the study protocol we defined treatment response as alcohol consumption within the British Medical Association boundaries (no more than 14 standard units for women, or 21 units for men, per week) [25], with an additional provision that participants did not present with a deterioration of more than 10% on the AUDIT [15], the Quality Of Life Scale [26], or the Global Severity Index of the Brief Symptom Inventory [27]. In other words, any such deterioration precluded our definition of treatment response. Positive treatment response, meeting these criteria, should be interpreted as desirable outcome of treatment and covers the wider aspects of problem drinking beyond drinking quantities only.

The central outcome for the cost utility analysis was the number of quality-adjusted life-years (QALYs) as calculated with the 5-dimensional EuroQol (EQ-5D) [28] using Dolan’s UK tariff to obtain preference-based utilities [29]. We calculated QALYs taking into account the 6-month timeframe of this study.

### Data Analysis

We carried out all analyses on an intention-to-treat basis. Missing observations in costs and effects data were handled using multiple imputation. The multiple imputation software package Amelia II [30] for R [31] has been found to yield the most accurate results in the type of data used in this study [32]. Amelia II takes into account the covariance structure between all variables, as opposed to some other approaches that require explication of covariates. Using this software, we imputed the original dataset 5 times, a sufficient number of imputations according to Rubin’s analysis of the required number of imputed sets needed for the missingness rates in the sample analyzed [33]. Analyses were performed on each of these 5 datasets separately, and the outcomes were then combined using Rubin’s rules for combining estimates obtained from multiply imputed datasets [33] for means. For medians and for the figures, we combined (appended) the data from the imputations. The relative attrition between the trial arms was low: IT:IS = ((41/68) / (39/68)) = 1.05 (Figure 1). Analyses were performed using SPSS 17.0 (IBM Corporation, Somers, NY, USA) and R 2.11.0 [31] software.

### Cost and Effect Data

We analyzed cost and effect data according to methods suggested by Drummond and colleagues [34]. For all participants, we multiplied units of health care (eg, sessions, contacts), time investments, and productivity losses by their associated costs. Differences in costs and effects between IT and IS were calculated at the 6-month follow-up measurement, because randomization had resulted in sufficient comparability across conditions at baseline (Table 2).

**Table 2:** Baseline characteristics of participants in Internet-based therapy (IT) and Internet-based self-help (IS).

Characteristic		IT (n = 68)	IS (n = 68)	*t*_134_/ Fisher exact test	*P *value
Women, n (%)		35 (51%)	35 (51%)	0.00	1.00
Age (years), mean (SD)		41.9 (10.1)	41.1 (9.6)	0.49	.63	
**Education, n (%)** ^a^				4.49	.10
	Low	2 (3%)	7 (11%)		
	Medium	24 (38%)	30 (46%)		
	High	38 (59%)	29 (44%)		
Employed, n (%)		58 (85%)	55 (82%)	0.25	.65
**Residential urbanization level, n (%)**				0.74	.75
	Low	9 (13%)	6 (9%)		
	Medium	21 (31%)	22 (32%)		
	High	37 (55%)	40 (59%)		
AUDIT^b ^composite score, mean (SD)		18.8 (4.8)	19.6 (5.6)	0.98	.33
Duration of alcohol problems (years), mean (SD)		5.2 (5.7)	5.4 (5.7)	0.23	.82
Drinks per week, mean (SD)		45.2 (26.3)	43.4 (24.0)	0.38	.71
EQ-5D^c ^score		0.79 (0.20)	0.80 (0.18)	0.32	.75
Work absenteeism^d^		756 (2289)	1863 (6983)	1.24	.22
Work presenteeism^d^		1137 (2386)	794 (1922)	0.78	.44

^a ^Classified according to Statistics Netherlands (CBS) and International Standard Classification of Education 1997.

^b ^Alcohol Use Disorders Identification Test [15].

^c ^5-dimensional EuroQol instrument, score calculated using the measurement and valuation of health (MVH-A1) algorithm from Dolan [29].

^d ^Averaged costs over the 6 months preceding baseline measurement.

### Bootstrapping

We extracted 1000 nonparametric bootstrapped [35] samples (n = 68 per trial arm) from each of the 5 multiply imputed datasets. For each of these 5 × 1000 bootstrapped samples, we calculated the incremental costs, incremental effects, and incremental cost effectiveness ratio (ICER). This ICER was calculated as follows: ICER = (C_IT _– C_IS_)/(E_IT _– E_IS_), where C is costs, E is effects, and the subscripts IT and IS refer to the two interventions. As effects, we used two outcome measures: (1) proportion of treatment responders, and (2) QALYs.

### Cost Effectiveness Plane

**Figure 2:**
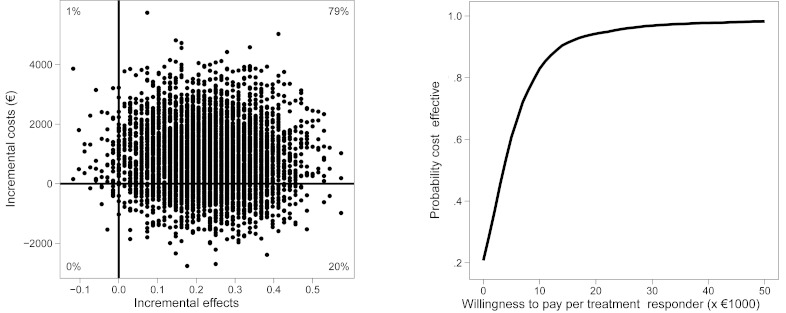
Cost effectiveness plane (left) and cost effectiveness acceptability curve (right) with treatment response as the effect measure.

The resulting 1000 ICERs per dataset were used for further calculations and plotted on the cost effectiveness plane [36] (Figure 2). The reference intervention (IS) is positioned in the origin of the cost effectiveness plane. The horizontal axis indicates differences in health gains between IT and IS and the vertical axis represents differences in costs. Along the horizontal and vertical axis, Figure 2 is divided into quadrants, each with a specific interpretation. ICERs that fall in the upper right quadrant indicate that IT generated better health for additional costs; the lower left quadrant indicates a reduction in health gains for fewer costs. In the upper left quadrant, IT is dominated by IS, as poorer health outcomes were obtained at additional costs. In the lower right quadrant, IT dominates IS with better health outcomes for fewer costs. The median values of the bootstrapped ICERs are presented in the Results section.

### Cost Effectiveness Acceptability Curve

**Figure 3:**
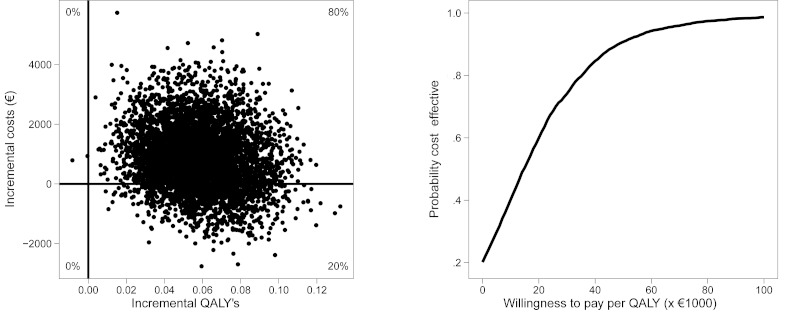
Cost effectiveness plane (left) and cost effectiveness acceptability curve (right) with quality-adjusted life-year (QALY) as the effect measure.

Based on the distribution of the ICERs over the cost effectiveness plane, cost effectiveness acceptability curves [37] were drawn (Figure 3). The cost effectiveness acceptability curves show the probability that IT is more cost effective than IS as a function of the willingness to pay (WTP) for 1 additional unit of effect (1 treatment responder or 1 QALY). At a probability of 0.5 on the vertical axis, the indifference point is reached. Above this indifference point, IT is to be preferred over IS with regard to cost effectiveness. WTP is an unknown quantity and therefore presented as a series of increments on the horizontal axis.

### Sensitivity Analysis

To test the robustness of the economic evaluation, we performed a sensitivity analysis in which we varied the most relevant cost drivers. First, the cost effectiveness analysis was replicated from the health care provider perspective, including only health care costs in the analysis. In other alternative scenarios, the influence of the most influential cost drivers (ie, intervention costs and productivity costs) was explored. These costs drivers were raised and lowered independent of each other, in order to test the influence of adjustments on the median ICER and the likelihood that IT is more cost effective than IS.

## Results

### Participants

Of the 136 participants included in this cost effectiveness analysis, 68 were randomly assigned to IT and 68 to IS. The participants (n = 70, 51% women) were a mean of 41.5 (SD 9.83) years old (Table 2). Reported drinking frequencies and AUDIT composite score indicated harmful drinking behavior at baseline. None of the baseline characteristics differed markedly between the groups.

### Costs

Per-participant costs in IT and IS, and bootstrapped incremental costs are presented in Table 3. All costs were estimated for the 6-month period between baseline and follow-up. Total intervention costs for IT and IS were on average €283 and €12, respectively (Table 1). Software development costs were, contrary to what is sometimes thought, prospective costs and not sunk costs. Complex software products such as the e-mental health interventions in this study needed continuous updates, bug fixes, security adjustments, and improvements to make sure they functioned with more recent browsers, operating systems, etc. Therefore, development costs were to a large extent running costs, and it is common in eHealth cost effectiveness studies to include these costs in the cost analysis (eg, [12-14]). For both groups, the largest cost drivers at follow-up were costs due to productivity losses (IT: €1331; IS: €886). The difference between IT and IS in mean costs of work absenteeism and presenteeism seems considerable and relevant (though not statistically significant), but must be seen in light of the actual number of participants who reported these costs. At baseline, 8 participants in IT and 12 participants in IS reported absenteeism; for presenteeism at baseline, these counts were 21 for IT and 15 for IS. The number of participants reporting absenteeism 6 months later dropped to 5 for IT and 2 for IS; presenteeism was reported by 7 in IT and 5 in IS. Total average societal costs for IT, €2010, were higher than the average €1120 for IS. The median difference of the societal costs between IS and IT was €845, which means that IT was more costly than IS from a societal perspective. The main incremental cost drivers were productivity costs and intervention costs.

**Table 3:** Costs and increments in the 6-month period preceding follow-up of the Internet-based therapy (IT) and Internet-based self-help (IS) groups^a^.

Cost type		IT		IS		Bootstrapped difference	
		Mean	SD	Mean	SD	Median	95% CI^b^
**Intervention costs**							
	Therapist labor	241	236	0	0	240	187–296
	Software development	23	0	5	0	18	18–18
	Software/hardware service	15	0	2	0	12	12–12
	Software overhead	4	0	4	0	0	0–0
	Total intervention costs	283	236	12	0	271	217–327
Participant time investment costs		95	103	22	37	72	48–99
**Productivity costs**							
	Work absenteeism	1114	5704	536	3800	555	–967 to 2234
	Work presenteeism	217	847	350	1637	–119	–609 to 256
	Total productivity costs	1331	5774	886	4215	417	–1215 to 2208
**Societal costs**							
	Additional societal costs^c^	301	1305	200	953	94	–275 to 499
	Total societal costs	2010	7141	1120	5167	845	–1157 to 3048
Treatment response (proportion)		0.53		0.29		0.24	0.07–0.38
EQ-5D^d ^score		0.89	0.20	0.78	0.34	0.12	0.05–0.18
ICER^e ^treatment response						3683	–5703 to 20,366
ICER QALY^f^						14,710	–18,337 to 71,664

^a ^All costs have been rounded for presentation in this table, and may therefore not add up exactly.

^b ^Confidence interval.

^c ^An estimation of real costs, based on Rehm et al [2], and includes additional health care resource costs and law-enforcement costs.

^d ^5-dimensional EuroQol instrument, score calculated using the measurement and valuation of health (MVH-A1) algorithm from Dolan [29].

^e ^Incremental cost effectiveness ratio.

^f ^Quality-adjusted life-year.

### Effects

Table 3 shows the proportion of favorable treatment response and the EQ-5D scores. In IT, 36 / 68 = 0.53 responded well to treatment after 6 months; in IS this was 20 / 68 = 0.29. Incremental effectiveness of IT compared with IS was therefore 0.53 - 0.29 = 0.24. Dolan’s [29] EQ-5D scores for IT and IS at 6 months, which can be used for cost utility analysis, were 0.89 and 0.78, respectively. The incremental utility gain of IT relative to IS can thus be calculated as 0.89 - 0.78 = 0.12. Considering the 6-month timeframe of this study, and the fact that mortality of participants in this study was zero, the number of incremental QALYs gained with 1 IT intervention compared with 1 IS intervention can be calculated as 0.12 * (6 / 12) = 0.06 [34].

### Cost Effectiveness Analysis

By dividing the incremental costs by the incremental effects, the mean ICER of IT compared with IS from the societal perspective is calculated as €845/0.24 = €3521 for 1 additional treatment responder, 6 months after inclusion. Using the bootstrapping procedure, we estimated the median ICER to be €3683. In the cost effectiveness plane (Figure 2, left), each dot represents a bootstrapped mean ICER. By calculating the proportion of simulated ICERs in each of the 4 quadrants, we found that IT had a 79% probability of leading to additional effects at additional costs relative to IS. A total of 20% fell into the dominant quadrant, indicating that there was a 20% likelihood that IT led to additional effects at lower societal costs (Table 4). The WTP at 50% was €3683 per additional treatment responder. Above a WTP of €3683 per additional treatment responder, IT must be considered cost effective in comparison with IS.

### Cost Utility Analysis

The mean incremental societal costs for 1 additional QALY gained by IT compared with IS were €845 / 0.06 = €14,083. The median ICER for 1 extra QALY was estimated too be €14,710. From Figure 3 (left) it becomes clear that there was an 80% probability that IT led to a better QALY health gain at additional costs, while 20% of the ICERs fell into the dominant quadrant. The cost effectiveness acceptability curve (Figure 3, right) suggests that at a WTP of €20,000 for 1 additional QALY, the probability that IT was more cost effective than IS was at 60% (Table 4). At a WTP of €14,710 or more for 1 additional QALY, IT must be considered cost effective in comparison with IS.

### Sensitivity Analysis

In Table 4, alternative costing scenarios are explored. From the health care provider perspective, the median ICER was €1157 per additional treatment responder, or €4693 per additional QALY. In other alternative costing scenarios, the main incremental cost drivers (intervention costs, costs due to productivity losses, and associated societal costs) were adjusted over a range of ±60%, in order to explore their impact on the ICERs. The results for ±40% adjustments are presented in Table 4. We found that ICERs were more sensitive to changes in productivity losses than to changes in intervention costs. Adjustments in both intervention and productivity costs led to the largest changes in ICERs. In all sensitivity scenarios in Table 4, the point of indifference from the cost effectiveness perspective between IT and IS was below a WTP of €20,000 per QALY, indicating that in any alternative scenario in this table, IT would be preferred over IS at a WTP of €20,000 or more per QALY.

**Figure 4:**
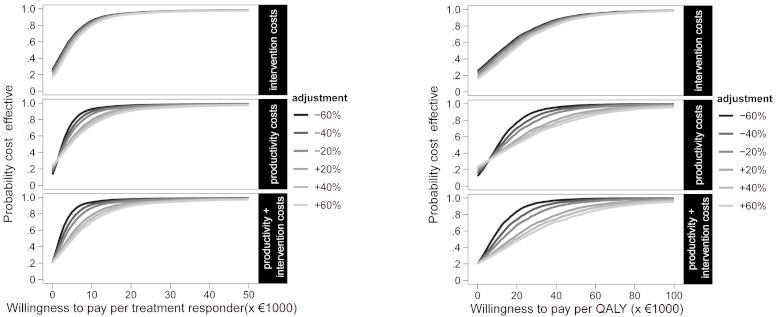
Cost effectiveness acceptability curve after sensitivity analyses with treatment response (left) and quality-adjusted life-year (QALY) (right) as effect measures.

**Table 4:** Cost effectiveness analysis of base case, health care provider perspective, and additional sensitivity analyses.

Cost drivers		Base case: societal	Alternative case: health care provider	Sensitivity analyses					
				I^a ^–40%	I +40%	P^b ^–40%	P +40%	I and P –40%	I and P +40%	
Incremental costs (median)		845	271	739	954	681	1012	573	1120
**Treatment response**									
	Incremental effects (median)	0.24	0.24	0.24	0.24	0.24	0.24	0.24	0.24
	ICER^c ^(median)	3683	1157	3187	4172	2977	4387	2494	4868
	ICER (95%low)	–5703	665	–6441	–5050	–3227	–8313	–3821	–7576
	ICER (95%high)	20,366	3722	19,410	21,409	14,724	25,979	13,738	26,957
	WTP^d ^€4000	53%	95%	57%	50%	62%	48%	66%	46%
	WTP €8000	76%	98%	78%	74%	85%	69%	87%	67%
	WTP €12,000	87%	99%	89%	86%	92%	82%	93%	80%
	Upper right quadrant	79%	99%	76%	82%	83%	76%	79%	79%
	Upper left (inferior) quadrant	1%	1%	1%	1%	1%	1%	1%	1%
	Lower left quadrant	0%	0%	0%	0%	0%	0%	0%	0%
	Lower right (dominant) quadrant	20%	0%	23%	17%	16%	22%	20%	20%
**QALYs^e^**									
	Incremental QALYs (median)	0.06	0.06	0.06	0.06	0.06	0.06	0.06	0.06
	ICER QALY (median)	14,710	4693	12,932	16,584	11,876	17,683	9946	19,436
	ICER QALY (95%low)	–18,337	2783	–20,177	–16,241	–10,291	–26,220	–12,282	–24,352
	ICER QALY (95%high)	71,664	10,848	67,913	75,671	52,202	91,101	48,403	94,958
	WTP €10,000	40%	95%	45%	36%	44%	38%	50%	35%
	WTP €20,000	60%	99%	64%	57%	70%	54%	74%	51%
	WTP €40,000	85%	100%	87%	83%	93%	77%	94%	74%
	Upper right quadrant	80%	100%	76%	83%	84%	77%	80%	80%
	Upper left (inferior) quadrant	0%	0%	0%	0%	0%	0%	0%	0%
	Lower left quadrant	0%	0%	0%	0%	0%	0%	0%	0%
	Lower right (dominant) quadrant	20%	0%	23%	17%	16%	23%	20%	20%

^a ^Intervention costs.

^b ^Productivity costs.

^c ^Incremental cost effectiveness ratio.

^d ^Willingness to pay.

^e ^Quality-adjusted life-year.

## Discussion

### Main Findings

In this cost effectiveness analysis, we found that the IT intervention led to almost double (0.53 versus 0.29) the number of treatment responders at 6 months, which was achieved at an incremental cost of €845 (equivalent to US $1008, based on purchasing power parity for the reference year 2010 [38]). Hence, 1 additional treatment responder for IT compared with IS was achieved at a median incremental cost of €3683 (US $4394). IT led to better EQ-5D health-related quality of life and 1 additional QALY was gained at a median incremental cost of €14,710 (US $17,548). At a WTP of €20,000 (US $23,859) [39] for 1 QALY gained, IT had a 60% likelihood of being more cost effective than IS. These results are somewhat sensitive to large adjustments in societal costs, but the alternative cost scenarios were also in favor of IT, assuming a WTP of, but not more than, €20,000 (US $23,859) for gaining an additional QALY.

### Implications

The maximum WTP per QALY is a matter of debate, but the figure of €20,000 is conservative compared with the World Health Organization’s recommendation of a maximum cost per QALY of 3 times the gross domestic product per capita (€88,000 for the Netherlands in 2010) [40]. The main findings of this study indicate that, from a cost effectiveness perspective, IT could be offered rather than IS, but the difference (60%) is not very large. In a conventional hypothesis-testing statistical approach, this difference would not be considered statistically significant, although such an approach is uncommon in cost effectiveness analyses alongside randomized controlled trials [34].

It is clear that the costs of providing IS from a health care provider perspective are only a fraction of those of providing IT. Alternatively, a stepped-care approach could be proposed, in which a client is referred to IS first and then is referred to IT in a second step when desirable results have not been achieved after IS.

### Previous Studies

Smit and colleagues [14] found that Internet-based alcohol self-help has a 73% probability of dominating from a cost effectiveness point of view compared with a text-only information leaflet. They found a negative ICER (US -$13,950, ie, a cost saving), mainly due to lower costs of productivity losses in the self-help intervention. In the current study, we did not find a relative reduction in productivity losses between the two active interventions. Solberg and colleagues [41] reviewed cost effectiveness studies from the health care provider perspective for brief (non-Internet-based) alcohol interventions. The number of QALYs gained was found to be highly sensitive to the effectiveness of counseling. Screening and brief counseling compared with no intervention had an overall ICER of US $1755 (in year 2000 terms) per QALY gained. Compared with this figure, the median incremental cost per QALY for IT compared with IS is less favorable. However, the lower cost per gained QALY can be expected when a comparison between an active intervention and no intervention is made.

### Strengths and Limitations

A limitation of this study stems from the generalizability of the cost data. The reported software costs were based on actual cost records, which may be different in other settings. To estimate full societal costs, we measured productivity loss cost data using the SF-HLQ, but we estimated health care costs other than the focal intervention and for law enforcement based on Rehm and colleagues [2]. Two potential issues arise: (1) data presented in Rehm et al [2] are not limited to harmful users, and (2) not all costs included as societal costs may be drinking related. Because additional health care costs and law-enforcement costs depend on productivity costs, we leveraged a potential bias in the measurement of productivity costs into these costs.

We collected data on productivity losses using the SF-HLQ, aiming at a 2-week period before data collection. Subsequently, we extrapolated the calculated costs of productivity losses. An alternative approach to calculating absence days could have been applied by (1) retrospectively asking participants about their work absenteeism in the previous 3 or 6 months, and thus collecting an alternative measure of absenteeism based on which we could have validated our extrapolation approach, or (2) measuring absenteeism, presenteeism, and the main clinical end points more frequently, in order to have more data on which to base the extrapolation. This would, however, have increased the research burden on our study participants, but it would presumably also have led to more sound data on costs (and effects). To assess the robustness of the results in terms of deviations from the calculated costs, we performed sensitivity analyses, plus an additional analysis in which we took into account only the intervention costs (health care provider cost perspective).

Another limitation was the time horizon in this analysis, which was restricted to 6 months. It is very possible that clinical effects were maintained after 6 months, although they may diminish over time. The same may be true for losses or gains in (productivity) costs. We have, however, not modeled the possible developments of effects and costs beyond the 6 months for which we have empirical data. This limits the time horizon and may jeopardize informed decision making when considering long-term effects.

By collecting patient-level cost data alongside a pragmatic randomized controlled trial, this study has both good comparability of the populations in the two interventions as a consequence of random allocation, and acceptable external validity as a result of the pragmatic approach [8]. Missing observations were multiply imputed; failing to account for missing costs data properly can produce biased results [42-44]. We subjected our base-case results to cost adjustments in a sensitivity analysis. Our main findings remained stable under alternative costing scenarios. At a WTP of €20,000 per QALY gained, IT offers outcomes for money equal to or better than those obtained with IS, and might therefore be considered as a possible treatment option, either as first-line treatment in a matched-care approach or as second-line treatment in the context of a stepped-care approach.

## Abbreviations

AUDIT: Alcohol Use Disorders Identification Test
EQ-5D: 5-dimensional EuroQol
ICER: incremental costeffectiveness ratio
IS: Internet-based self-help
IT: Internet-based therapy
QALY: quality-adjusted life-year
SF-HLQ: Short Form-Health and Labor Questionnaire
WTP: willingness to pay
